# Formation of oxygen vacancies and Ti^3+^ state in TiO_2_ thin film and enhanced optical properties by air plasma treatment

**DOI:** 10.1038/srep32355

**Published:** 2016-08-30

**Authors:** Bandna Bharti, Santosh Kumar, Heung-No Lee, Rajesh Kumar

**Affiliations:** 1 Jaypee University of Information Technology, Waknaghat, Solan-173234, H.P., India; 2 School of Materials Science and Engineering, Gwangju Institute of Science and Technology (GIST), 123 Cheomdangwagi-ro Buk-gu, Gwangju, 61005, South Korea; 3 Gwangju Institute of Science and Technology (GIST), 123 Cheomdangwagi-ro Buk-gu, Gwangju, 61005, South Korea

## Abstract

This is the first time we report that simply air plasma treatment can also enhances the optical absorbance and absorption region of titanium oxide (TiO_2_) films, while keeping them transparent. TiO_2_ thin films having moderate doping of Fe and Co exhibit significant enhancement in the aforementioned optical properties upon air plasma treatment. The moderate doping could facilitate the formation of charge trap centers or avoid the formation of charge recombination centers. Variation in surface species viz. Ti^3+^, Ti^4+^, O^2−^, oxygen vacancies, OH group and optical properties was studied using X-ray photon spectroscopy (XPS) and UV-Vis spectroscopy. The air plasma treatment caused enhanced optical absorbance and optical absorption region as revealed by the formation of Ti^3+^ and oxygen vacancies in the band gap of TiO_2_ films. The samples were treated in plasma with varying treatment time from 0 to 60 seconds. With the increasing treatment time, Ti^3+^ and oxygen vacancies increased in the Fe and Co doped TiO_2_ films leading to increased absorbance; however, the increase in optical absorption region/red shift (from 3.22 to 3.00 eV) was observed in Fe doped TiO_2_ films, on the contrary Co doped TiO_2_ films exhibited blue shift (from 3.36 to 3.62 eV) due to Burstein Moss shift.

Among various metal oxide semiconductors, TiO_2_ is considered as a prime candidate due to its many peculiar properties^1^,^2^ for diverse applications. It is the most suitable candidate for photocatalytic applications due to its biological and chemical inertness, strong oxidizing power, non-toxicity and long term stabilization against photo and chemical corrosion^3^. The films of TiO_2_ have valuable applications in LEDs, gas sensors, heat reflectors, transparent electrodes, thin film photo-anode to develop new photovoltaic, photo-electrochemical cells, solar cells and water splitting^4^,^5^,^6^,^7^,^8^,^9^,^10^. In anodic applications, it is a preferred material because of its low density/molar mass and structural integrity over many charge and discharge cycles^11^. However, the efficiency of pure TiO_2_ is substantially low because of its wide band gap and fast recombination of photo-generated electrons and holes. The key issue to improve the performance of TiO_2_ relies on efficient light harvesting, including the increase of its photo-efficiency and expansion of photo-response region, and to ensure efficient number of photo-generated electrons and holes reaching to the surface before their recombination. In order to meet these desired performances the bands structure modification of TiO_2_ is preferred.

Generally, three fundamental approaches are implemented for band structure modification viz. doping with metallic/non-metallic elements or co-doping of metallic and non-metallic elements^1^,^12^,^13^,^14^, modification via introducing defects such as oxygen vacancies and Ti^3+^ in the band gap^15^,^16^, and surface modification by treatment methods^11^,^17^,^18^,^19^. In metallic doping, among the range of dopants such as Ni, Mn, Cr, Cu, Fe etc.^3^,^20^,^21^,^22^,^23^, the Fe is found most suitable due to its half filled electronic configuration. Similarly, from non-metallic dopants S, C, F, N etc.^24^,^25^,^26^,^27^, the N is preferred. In the case of metallic dopants, there are some contradictory reports that show disadvantages of thermal and chemical instability of TiO_2_. Also, their high doping although enhances the band gap but at the same time reduces optical/photocatalytic activity because of increasing carrier recombination centers^28^,^29^,^30^,^31^. What is the mechanism of observed photo-response of doped/modified TiO_2_; it is still a question, however a generally accepted concern states that the photo absorption of a material is explained better by introducing the defects in the lattice of TiO_2_. For example, Ti^3+^ and oxygen vacancies^32^ create trap centers, rather than the recombination centers unlike the high doping case, and results in the variation of band gap of pristine TiO_2_.

On the other hand, surface modification methods including surface hydrogenation^33^, vacuum activation^32^ and plasma treatment^34^ are also practiced. In the hydrogenation method, the surface of TiO_2_ is terminated with hydrogen leading to an enhanced photocatalytic activity^35^ in visible region; however, it is still unknown that how does the hydrogenation modify a surface to enhance its optical performance (photocatalytic activity)^36^. The drawback of the hydrogenation method is that it requires high temperature and the obtained TiO_2_ sample/film are black^35^, which makes the films unable for many optoelectronic applications, such as a transparent electrode in optoelectronic devices. Both the vacuum activation and plasma treatment methods create highly stable Ti^3+^ and oxygen vacancies^32^,^34^. In vacuum activation method, the sample may exhibit higher absorption intensity but it appears brown in color^35^, that makes it unable for transparent electrode applications. Finally, in case of plasma treatment methods, generally hydrogen gas is used to create Ti^3+^ and oxygen vacancies in TiO_2_, but it is always avoidable to use such a hazardous and expensive gas. Except hydrogen there are few reports on the use of argon^37^, oxygen^38^ and nitrogen plasma^39^ for surface modification of TiO_2_. We know that the implementation of gas in the treatment chamber may be hazardous and cost effective; therefore, it is always required to avoid the use of hazardous gas, and to implement a simple and low cost approach to meet the requirements. In this regard, treatment by air plasma may be an effective approach. However, to the best of our knowledge there is no report on the application of air plasma for the surface modification of TiO_2_ film.

In this report, the band structure modification of thin transparent films of TiO_2_ was done by implementing simply the air plasma and thus creating Ti^3+^ and oxygen vacancies in TiO_2_ films. The effect of air plasma treatment was studied in conjunction with metallic doping. First, Fe and Co doped TiO_2_ thin films were formed on glass substrate, which were subsequently treated in air plasma. Considering the drawback of high metallic doping (formation of recombination centers), in this study, a moderate amount of dopants were used to enhance the optical properties of TiO_2_ thin film and thereafter the air plasma was applied to enhance them further. The moderate amount of metallic dopant not only favors the separation of electrons and holes but also narrows the band gap of TiO_2_^3^. We observed that simultaneous effect of the joint approaches increases photo absorbance as well as expends photo response region of the films towards both the visible and UV spectrum. The doped films of TiO_2_ were treated in plasma with varying treatment time. The moderate doping of Fe and Co elements reduces band gap minutely in both the cases, but when treated with air plasma a significant change in the optical properties was observed due to the formation of Ti^3+^ and oxygen vacancies in the band gap.

## Results and Discussion

**Figure 1:**
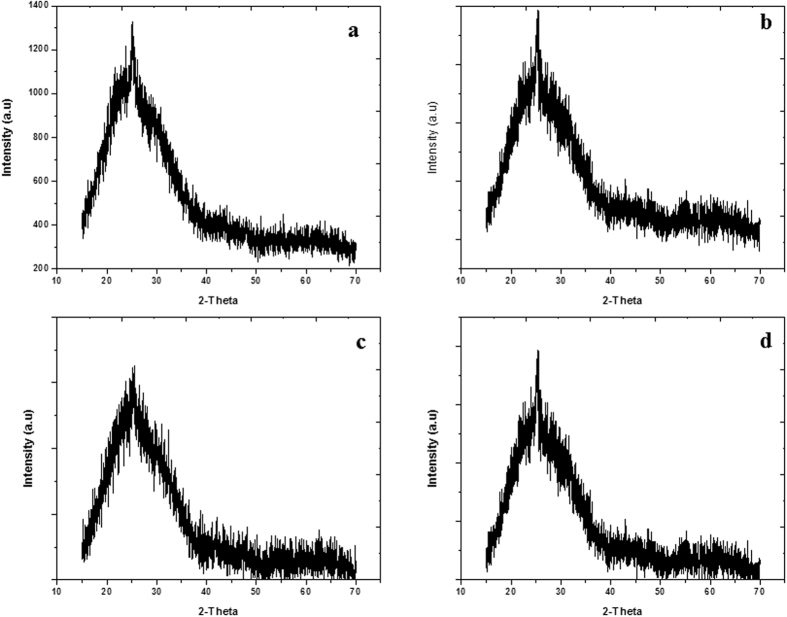
X-ray diffraction spectra of **(a)** Fe doped/untreated TiO_2_ film; plasma treatment time 0 second, **(b)** Fe doped/treated TiO_2_ film; plasma treatment time 60 second, **(c)** Co doped/untreated TiO_2_ film; plasma treatment time 0 second and **(d)** Co doped/treated TiO_2_ film; plasma treatment time 60 second.

After fabricating, the thin films of pure TiO_2_, Fe and Co doped TiO_2_ were treated in air plasma for 0, 10, 30 and 60 seconds, which were analyzed for surface morphology and crystal structure variations using SEM (see Supplementary Information; Figure S1) and XRD. Here we show XRD pattern of doped thin films for extreme treatment time 0 and 60 seconds (for XRD spectra of samples treated at other treatment time, please see Supplementary Information; Figure S2). Figure 1(a,b) represents XRD pattern of Fe doped, and Fig. 1(c,d) represents XRD pattern of Co doped TiO_2_ thin films for 0 (untreated) and 60 seconds of plasma treatment time. Since there is no detection of Fe and Co signals, it indicates that all the Fe and Co ions in the respective samples gets incorporated into the structure of TiO_2_ by replacing some of Ti ion, and occupying the interstitial sites^40^.

Absence of sharp peak in XRD patterns represents amorphous phase of TiO_2_ thin films^41^. After plasma treatment 2*θ* angle and FWHM of the peaks remain almost unchanged, indicating negligible effect on the film structure. XRD indicates that plasma treatment does not create any change in the crystal structure of Fe and Co doped TiO_2_ thin films. The obtained low signal-to-noise ratio in the above XRD spectra is due to the low crystallinity of the films and small crystallite size; such observations have been reported by others as well^42^.

The presence of atomic percentage of the dopants in TiO_2_ thin films was detected by EDX signals (see Supplementary Information; Figure S3). The EDX of Fe doped TiO_2_ film shows the atomic percentage of Fe, Ti and O as 1.66%, 12.93% and 85.41%, respectively, which closely matches to the stoichiometry of elements in Ti_0.95_Fe_0.05_O_2_. Similarly, in case of Co doped TiO_2_, the obtained atomic percentage of Co, Ti and O in EDX are 1.33%, 23.33% and 75.35%, respectively, which confirms the stoichiometry of elements of Ti_0.95_Co_0.05_O_2_ thin film.

Variation in optical properties of TiO_2_ thin films by doping and subsequent air plasma treatment was analyzed by UV-Vis spectrophotometer. The change in absorption edge and corresponding band gap is mentioned in Table 1. Pure TiO_2_ film (undoped and untreated) showed absorption edge at 367 nm and band gap 3.37 eV, whereas Fe doped TiO_2_ film showed a shift in the absorption edge to 385 nm, with a decreasing in the band gap to 3.22 eV. Similarly, Co doping shifts the absorption edge from 367 nm to 369 nm with a reduction in the band gap to 3.36 eV. The observed red shift in absorption edge and narrowing band gap in both dopants cases is similar to other reports on metallic doping^3^. In both the cases, samples were doped with a moderate (5%) concentration of Fe and Co forming Ti_0.95_Fe_0.05_O_2_ and Ti_0.05_Co_0.05_O_2_, respectively. We could have tuned the optical properties further by increasing the dopant concentration but that would form recombination centers^28^; therefore, to avoid the formation of recombination centers, a further tuning in the optical properties was done by treating these moderately doped TiO_2_ films in air plasma. The films were treated in air plasma for treatment time (0, 10, 30 and 60 seconds), and investigated for the shift in absorption edge and band gap variation. With increasing treatment time, the absorption edge of Fe doped TiO_2_ films shifts continuously from 385 nm (for 0 seconds treatment time) to 413 nm (for 60 seconds treatment time), with a corresponding band gap change from 3.22 eV to 3.00 eV, showing a significant increase in the absorption region. In case of Co doped TiO_2_ films, the absorption edge shifts from 369 nm to 342 nm (for 60 seconds treatment time) with a corresponding band gap change from 3.36 to 3.62 eV, which shows an increase in the optical band gap/UV absorption region probably due to the Burstein-Moss effect^43^, explained latter.

From the Table, it is observed that the change in optical properties of TiO_2_ films appears at two levels; first by the doping of Fe and Co, and then by plasma treatment. However, here it should be noted that the change in the band gap due to the doping is smaller as compared to the subsequent band gap change by plasma treatment. While discussing the effect of doping on the change of band gap, we know that the reduction may take place due to either by the increasing grain size of highly crystalline sample^44^ or the formation of electronic energy levels within energy band gap^45^. In our study, since the XRD results showed the samples to be amorphous, thus the first reason can be discarded. Therefore, Fe^3+^ and Co^2+^ ions substitute Ti^4+^ ions in TiO_2_ matrix and cause a change in the band gap by forming their mid gap energy levels in the respective samples along with the formation of Ti^3+^ and oxygen vacancies. The electronic transition from valance band to dopant level and then from dopant level to conduction band, and/or from valance band to oxygen level and then form oxygen level to Ti^3+^ level/dopant level effectively cause a red shift in the absorption edge, showing reduced band gap^46^,^47^,^48^. In many cases, the localized level of t_2g_ state of the doping element even lies in the middle of band gap (in case of, Cr, Mn or Fe as the doping materials), and at the top of the valance band (when Co is used as a dopant)^49^. Next, the variation in the absorption edge/band gap with plasma treatment time is due to the increase of Ti^3+^ and oxygen vacancies, detailed discussion is given under XPS studies in the following section.

**Figure 2:**
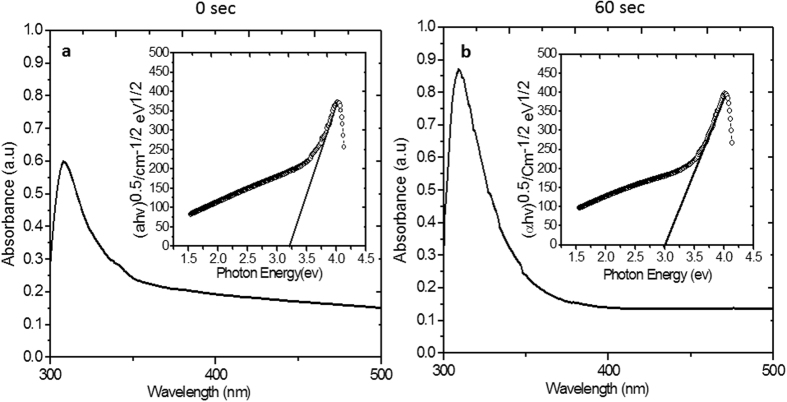
Optical absorption spectra and Tauc plot ((*αhv*)^1/2^ versus *hv* plot) in the inset for **(a)** Fe doped/untreated TiO_2_ film; plasma treatment time 0 second and **(b)** Fe doped/treated TiO_2_ film; plasma treatment time 60 second.

Figure 2 shows variation in the absorption spectra of Fe doped TiO_2_ thin film treated for 60 seconds of time (Fig. 2(b)) with respect to untreated one (Fig. 2(a)) (to see the increase in the absorption edge and reduction in band gap, please refer to Supplementary Information; Figure S4). There is a continuous change in the absorbance, absorption edge and band gap of the films with plasma treatment time. The absorbance of the film increased from 60% (untreated film) to 87% (treated for 60 seconds) along with a red shift in the absorption edge and band gap narrowing by 0.22 eV (Tauc plot shown in the inset of Fig. 2(b)). The band gap and absorption edge were estimated using the following equations^50^:


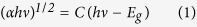



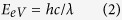


where *α* is absorption coefficient and *E*_*g*_ is band gap energy.

**Figure 3:**
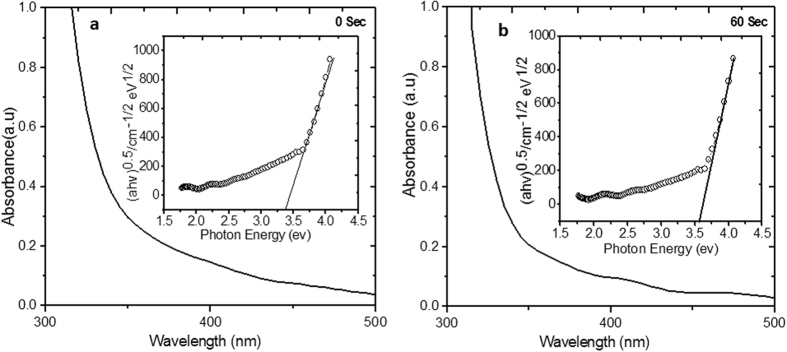
Optical absorption spectra and Tauc plot ((*αhv*)^1/2^ versus *hv* plot) in the inset for **(a)** Co doped/untreated TiO_2_ film; plasma treatment time 0 second and **(b)** Co doped/treated TiO_2_ film; plasma treatment time 60 second.

Similarly, the variation in absorption spectra of Co doped TiO_2_ thin film treated for 0 and 60 seconds is shown in Fig. 3(a,b) (details of other samples is given in Supplementary Information; Figure S5). In this case, doping shows a red shift due to the presence of Co levels in the energy gap of TiO_2_, whereas after plasma treatment the film shows continuous blue shift with increasing treatment time. This overall shift (due to treatment in plasma for 60 seconds) in the band gap is 0.26 eV. The observed blue shift can be explained by Burstein-Moss effect^43^, resulted by the change in the position of Fermi level into the conduction band. General equation representing enhancement in the band gap energy is given by:


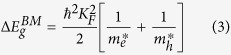


where 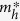 and 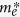 are the effective mass of hole and electron in the respective bands, and *K*_*F*_is Fermi wave vector. In our case, the shift of Fermi level into the conduction band leads to the energy band widening. Absorption edge shifts to shorter wavelength region due to the increase in the carrier concentration, which is discussed in XPS studies section.

**Figure 4:**
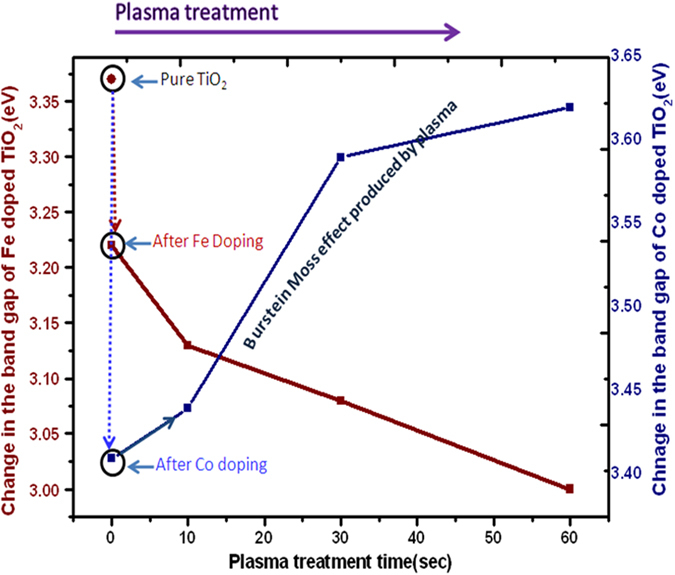
Plots for variation of optical band gap of Fe and Co doped TiO_2_ thin film with plasma treatment time.

The overall variation in the absorption edge and band gap of TiO_2_ thin film due to the doping (Fe and Co) and air plasma treatment is plotted in Fig. 4. In the plasma treatment region, a remarkable change in the band gap values can be observed with treatment time.

### XPS study

**Figure 5:**
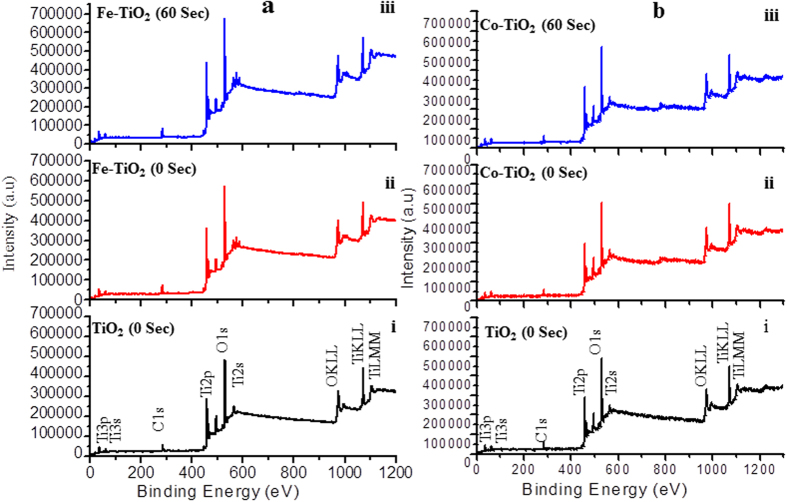
XPS survey spectra in **a**(i) pure TiO_2_ film indicating all the peaks of elements present in the sample, here the appeared carbon peak is instrumental impurity, **a**(ii) Fe doped/untreated TiO_2_ film; plasma treatment time 0 seconds, **a**(iii) Fe doped/treated TiO_2_; plasma treatment time 60 seconds, **b**(i) pure TiO_2_ film which is similar to **a**(i), and **b**(ii) Co doped/untreated TiO_2_ film; plasma treatment time 0 seconds, **b**(iii) Co doped/treated TiO_2_ film; plasma treatment time 60 seconds.

In order to understand the mechanism resulting the change in the band gap of Fe and Co doped TiO_2_ films with plasma treatment time, the films were investigated by XPS. The XPS being surface sensitive technique provides information about the change in chemical state of film constituting species. Here, the variation in the chemical state of elements ‘O’ and ‘Ti’ with plasma treatment time was analyzed in detail to correlate it with the observed variations in the band gap of the films. Figure 5(a,b) shows XPS survey spectra of untreated and plasma treated Fe and Co doped TiO_2_ thin films, respectively. In these spectra, C1s is probably an instrumental impurity. The intensities of O1s and Ti2p peaks increase with the increasing plasma treatment time, indicating an increase in these states with treatment time.

**Figure 6:**
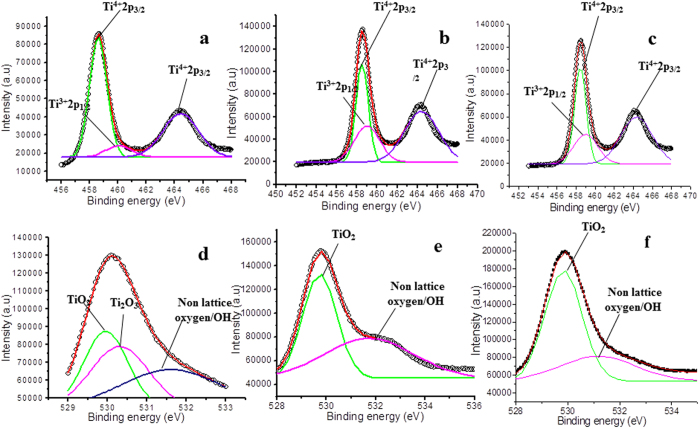
High resolution XPS spectra of Ti2p and O1s in **(a)** pure/untreated TiO_2_ film, **(b)** Fe doped/untreated TiO_2_ film; plasma treatment time 0 second, **(c)** Fe doped/treated TiO_2_ film; plasma treatment time 60 seconds, **(d)** O1s for pure/untreated TiO_2_ film, **(e)** O1s for Fe doped/untreated TiO_2_ film; plasma treatment time 0 second, and **(f)** O1s for Fe doped/treated TiO_2_ film; plasma treatment time 60 seconds.

Figure 6(a) shows high resolution XPS spectrum of pure TiO_2_ film. In this spectrum, the doublet ‘Ti2p_3/2_ (binding energy 458.6 eV) and Ti2p_1/2_ (binding energy 464.4 eV)’ arises from spin orbit-splitting. These peaks are consistent with Ti^4+^ in TiO_2_ lattice^51^. Also, the shoulder Ti2p_1/2_ at binding energy 460.2 eV is corresponding to Ti^3+^ ^52^ in Ti_2_O_3_. This indicates that both TiO_2_ and Ti_2_O_3_ are formed in the film (Without deconvolution, the XPS spectra are shown in Supplementary Figure S6). After doping with Fe, the high resolution XPS spectrum (Fig. 6(b)) shows a slight shift in the position along with a variation in the area of the original peaks. The peaks in the Fe doped samples are now located at binding energies 458.4 (Ti2p_3/2_), 464.3 eV (Ti2p_1/2_) and 459.0 eV (Ti2p_1/2_), respectively (see Supplementary Information; Table S1). The shift in the position of these peaks indicates an influence of Fe addition on the electronic state of Ti element; probably some of the Ti ions get substituted with Fe ions in the lattices. After doping, the area of Ti^3+^ peak increased by 81% and that of the peak Ti^4+^ decreased by 19%. The increase in the area of Ti^3+^ peak indicates that either Ti_2_O_3_ is formed in large amount or some mixed oxide structure with Fe (having oxidation state Ti^3+^) is formed after doping. Meanwhile, the decreasing area of Ti^4+^ indicates a reduction of TiO_2_ in the sample, and probably formation of Ti-O-Fe structure in the TiO_2_ lattice through the substitution of transition metal ions. Observed shift in the peaks also indicates interaction between Ti and Fe atoms and an overlapping of their 3d orbital^53^. This causes an electronic excitation from Fe to Ti in the optical absorption experiment, which shows a reduction in the band gap of Fe doped TiO_2_ film (as observed in the optical analysis).

After doping, the film was treated in air plasma. In the XPS results, only the sample which was treated for 60 seconds in plasma is demonstrated. The XPS shows a further increase in the peak corresponding to Ti^3+^ at 459.0 eV (Fig. 6(c)) and a decrease in the peak area of Ti^4+^. The change in stoichiometry was estimated by the change in the area of relative peaks. The peak area of Ti^3+^ increases by 20% and that of Ti^4+^ decreases by 12%. The increase in the peak area of Ti^3+^ indicates that after plasma treatment there is removal of oxygen from the lattice, which shows a relative increase in Ti^3+^ in the XPS spectrum. On the other hand decreasing peak area of Ti^4+^ is inferred due to the reaction of Ti^4+^ with electrons coming either from plasma or due to the formation of oxygen vacancies in the surface layer generated by the plasma treatment^41^. Now, as observed in optical analysis, the band gap of Fe doped films (3.22 eV) decreased to 3.00 eV (for 60 seconds of treatment time), this is correlated with the increasing career/electrons density due to plasma treatment. As we know that in the doped samples, the possible reasons of red shift/decreasing band gap is the introduction of donor states in the energy gap (here oxygen vacancies and Ti^3+^, Table 1). In the present case, the band gap decreases further with increasing treatment time, while the concentration of the dopant was kept constant, which is due to the change in the surface states of the constituents i.e. Ti element and oxygen vacancies.

Next, the O1s spectrum of pure TiO_2_ thin film is shown in Fig. 6(d), which is fitted with three peaks. The peaks at binding energies 529.9 eV, 530.3 eV and 531.3 eV are attributed to lattice oxygen, Ti_2_O_3_ and non-lattice oxygen^54^,^55^. Similarly, for the doped sample, O1s spectrum of Fe doped TiO_2_ thin film fitted with two peaks is shown in Fig. 6(e). In this spectrum, only two peaks at binding energies 529.8 eV, and 531.9 eV are observed which are attributed to lattice oxygen and surface adsorbed OH group, whereas the peak 530.3 eV corresponding to Ti_2_O_3_, disappears. This indicates that in the doping process TiO_2_ is formed along with some mixed oxide. Again, the change in stoichiometry was estimated by the change in area of relative peaks. In case of Fe doped TiO_2_ film, the area of the peak at 529.7 increases by 64% and that of the peak at 531.5 eV increases by 54%.

After plasma treatment, the binding energy of lattice oxygen (O in TiO_2_) shifts slightly from 529.8 eV to 529.7 eV (Fig. 6(f)), whereas its area increases by 35%. Also, the area of the peak at 531.5 eV (non-lattice oxygen/OH) increases by 15% (see Supplementary Information; Table S1). The increase in the area of non-lattice oxygen indicates the formation of oxygen vacancies in the lattice. This result is analogues to the XPS spectrum of Ti2p (Fig. 6(c)).

**Figure 7:**
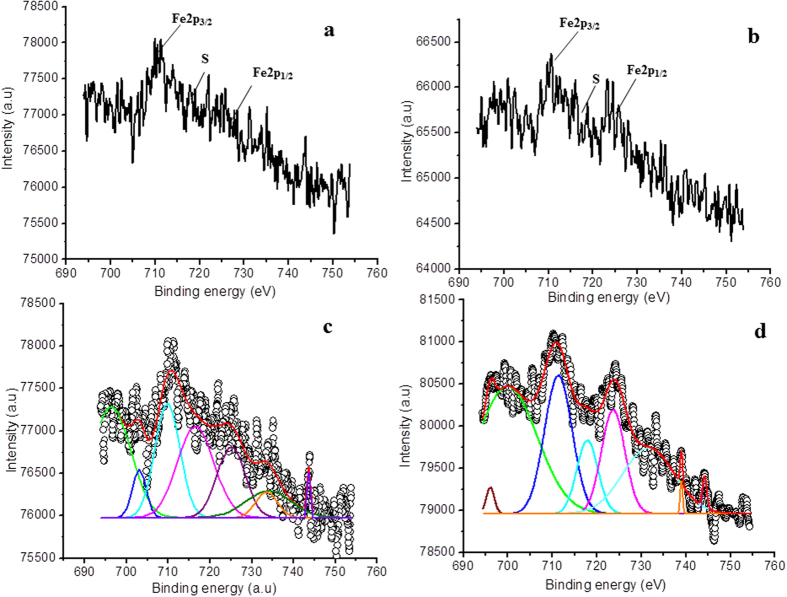
High resolution XPS spectra of Fe2p in **(a)** Fe doped/untreated TiO_2_ film; plasma treatment time 0 second, **(b)** Fe doped/treated TiO_2_ film; plasma treatment time 60 seconds, **(c**,**d)** are Gaussian fit of **(a**,**b)**.

Fe doping results in a minor shift in the binding energy, indicating that Fe ions are better dispersed in the substitutional sites of TiO_2_ lattice and produce more mixed oxide structure, probably Fe-O-Ti. Figure 7(a) shows high resolution XPS spectrum (for Fe2p_3/2_) of Fe doped TiO_2_ film. After plasma treatment, the high resolution XPS spectrum of Fe2p_3/2_ is shown in Fig. 7(b). These spectra are fitted with Gauss–peak shapes as shown in Fig. 7(c,d). The deconvoluted XPS spectrum of Fe2p_3/2_ (Fig. 7(c,d)) contains main peaks at 710.1 eV and 724.6.1 eV corresponding to Fe2p_3/2_ and Fe2p_1/2_, respectively (see Supplementary Information; Table S2). The appearance of these peaks supports the presence of Fe in Fe^3+^ ionic state^55^. Further, after plasma treatment the shift in the binding energy of Fe2p_3/2_ from 710.1 eV to 711.3 eV also indicates the presence of Fe^3+^ species, irrespective of the particular oxide (i.e., Fe_2_O_3_, Fe_3_O_4_, and FeOOH). Shake up satellite at 716.9 eV also supports that Fe is presented in Fe^3+^ state (oxide)^56^. These shake-up satellites are associated with Fe3d-O2p hybridization. Thus XPS analysis confirmed that Fe ions are doped into TiO_2_ matrix in the form of Fe-O-Ti. From the XPS analysis, we confirmed that by increasing the plasma treatment time the concentration of Ti^3+^ and oxygen vacancies also increases.

**Figure 8:**
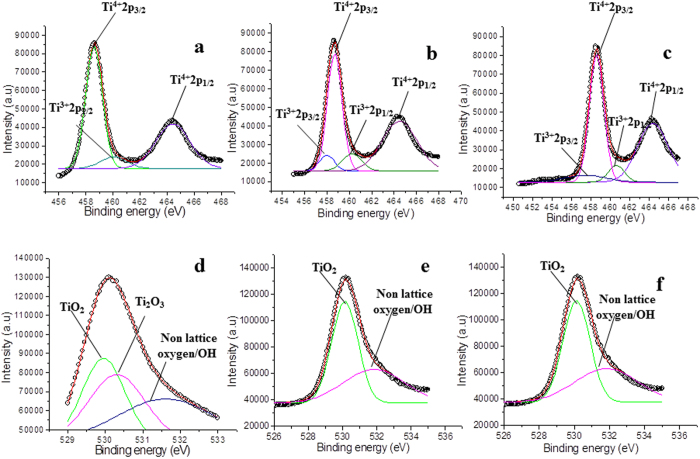
High resolution XPS spectra of Ti2p and O1s in **(a)** pure/untreated TiO_2_ film, **(b)** Co doped/untreated TiO_2_ film; plasma treatment time 0 second, **(c)** Co doped/treated TiO_2_ film; plasma treatment time 60 seconds, **(d)** O1s for pure/untreated TiO_2_ film, **(e)** O1s for Co doped/untreated TiO_2_ film; plasma treatment time 0 second, and **(f)** O1s for Co doped/treated TiO_2_ film; plasma treatment time 60 seconds.

The Co doped samples after treating in plasma show adverse effect on the band gap of the doped TiO_2_ film. In this case, band gap increases with the increasing treatment time as observed in optical studies. To investigate this divergent behavior, the samples were analyzed via XPS, Fig. 8 shows high resolution spectra. Figure 8(a) shows the XPS spectrum of pure TiO_2_, and Fig. 8(b) shows XPS for Co doped sample. As discussed above in the case of Fe doped sample, the XPS of pure TiO_2_ is also fitted with three peaks corresponding to titanium dioxide (Ti^4+^) and titanium sub oxide (Ti^3+^) in Ti2p_1/2_ and Ti2p_3/2_, respectively. These peaks are fitted as Ti^4+^2p_1/2_ at 464.4 eV, Ti^4+^2p_3/2_ at 458.6 eV, and Ti^3+^2p_3/2_ at 460.2 eV. The line separation between Ti2p1/2 and Ti2p3/2 is 5.8 eV, which is consistent with the standard binding energy of TiO_2_^51^. However, in this case the Ti2p spectrum (Fig. 8(b)) is fitted with four peaks as 464.4 for Ti^4+^2p_1/2_, 458.6 eV for Ti^4+^2p_3/2_, 460.4 for Ti^3+^2p_3/2_ and 457.9 eV for Ti^3+^2p_1/2_^57^, respectively (see Supplementary Information; Table S1). In comparison to the pure TiO_2_, the area of Ti^3+^ peak in Co doped TiO_2_ increases by 26%, while that of the peak Ti^4+^ decreases by 7%, indicating a reduction in the formation of TiO_2_, which is similar to the case of Fe doped samples.

After the plasma treatment (Fig. 8(c)), binding energies of the mentioned peaks are shifted slightly to the positions such as 464.3 eV (Ti^4+^2p_1/2_), 458.5 eV (Ti^4+^2p_3/2_), 460.6 eV (Ti^3+^2p_3/2_) and 457.4 eV (Ti^3+^2p_1/2_), respectively. The change in stoichiometry was estimated by the change in peak area of respective peaks.

After plasma treatment, while investigating for peak area, we observed that the peak area of Ti^3+^ increases by 30%, whereas the peak area of Ti^4+^ decreases by 12%. Again, this is expected due to the reaction of Ti^4+^ with the electrons coming either from plasma or due to the formation of oxygen vacancies in the surface layer by the plasma treatment. Further, the high resolution O1s XPS spectrum obtained for Co doped sample is shown in Fig. 8(d–f). The spectrum is fitted with three peaks i.e. 529.9 eV, 530.3 eV and 531.6 eV that correspond to lattice oxygen of TiO_2_, oxygen in Ti_2_O_3_ and non-lattice oxygen, respectively.

The change in stoichiometry was estimated by change in the peak area of relative peaks. With the doping of Co, the lattice oxygen (corresponding to TiO_2_) peak at 529.9 shifts to the position 530.3 eV, and the area of the peaks at 530.3 eV and 531.6 eV increases by 51% and 24%, respectively. The original peak at 530.3 eV (Fig. 8(d)) corresponding to Ti_2_O_3_ disappears after doping (Fig. 8(e)), which is due to the formation of mixed oxide structure. Further, with the increasing treatment time, the areas of the peaks at 530.3 eV and 531.6 eV ((Fig. 8(f)) also increases by 24% and 25%, respectively. (To explain in a more quantitative manner we have tabulated all the data in a table by comparing all the peaks at different plasma treatments time, see Supplementary Information; Table S1).

**Figure 9:**
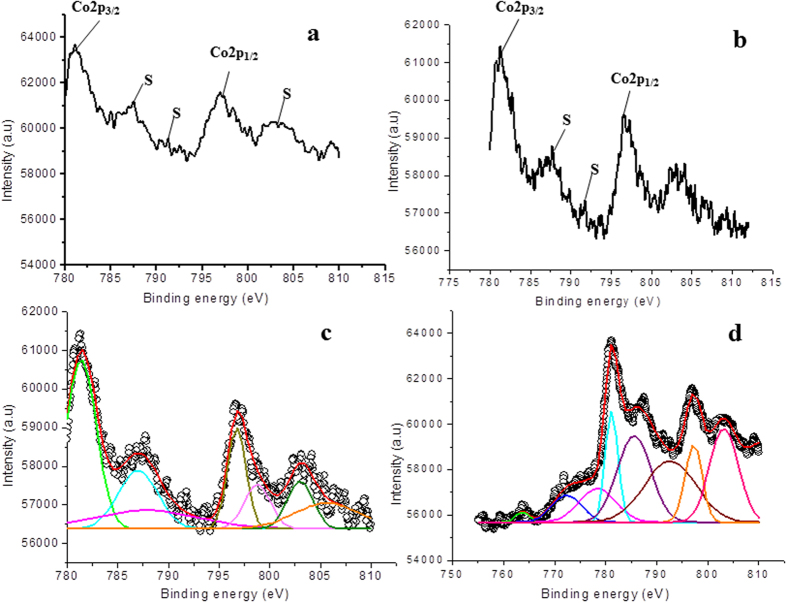
High resolution XPS spectra of Co2p in **(a)** Co doped/untreated TiO_2_ film; plasma treatment time 0 second, **(b)** Co doped/treated TiO_2_ film; plasma treatment time 60 seconds, **(c**,**d)** are Gaussian fit of **(a**,**b)**.

Next, Fig. 9(a) corresponds to high resolution XPS spectra of Co2p region of Co doped TiO_2_ thin films and Fig. 9(b) shows high resolution XPS spectra with plasma treatment. Figure 9(c,d) represent deconvoluted XPS spectra of doped TiO_2_ and plasma treated TiO_2_ thin films, respectively. The core level binding energies of peaks Co2p_1/2_ and Co2p_3/2_ are 796.9 eV and 781.0 eV, respectively. The satellite peaks at 787 eV and 802 eV reveal high spin Co(II) state with complex transitions^58^. These results are an indication that Co does not precipitate as metallic Co on the film surface. After plasma treatment, the satellites peaks shifts slightly to the 785.3 eV and 802.3 eV. Also, the binding energies of Co2p_1/2_ and Co2p_3/2_ are shifted to 796.6 eV and 781.2 eV, respectively (see Supplementary Information; Table S1). These spectra are typical of compounds containing high-spin Co^2+^ ions^59^,^60^, reveling the presence of CoO(Co^2+^), CoTiO_3_ (Co^2+^), Co_2_O_3_ (Co^3+^) or mixed valence Co_3_O_4_ (Co^2+^ and Co^3+^) in the surface. The presence of strong satellites indicates that Co atoms in the doped TiO_2_ film are in 2+ oxidation state, referring the possible formation of CoO or CoTiO_3_ inside the film.

**Figure 10:**
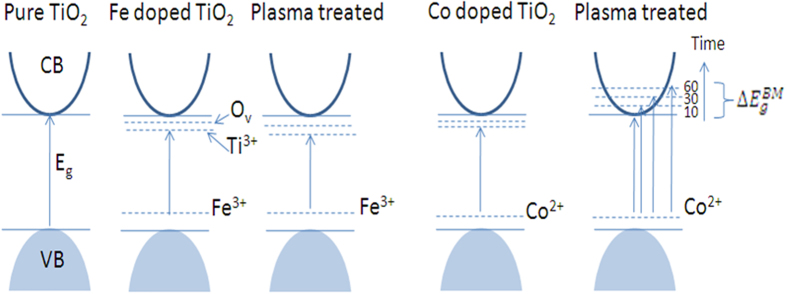
Schematic diagram of the energy levels of **(a)** pure/untreated TiO_2_ films, **(b)** Fe doped/untreated TiO_2_ film, **(c)** Fe doped/treated TiO_2_; for 60 seconds of treatment time, **(d)** Co doped/untreated TiO_2_ film, **(e)** Co doped/treated TiO_2_ film; for 10, 30 and 60 seconds of treatment time, indicating Burstein Moss effect. (O_v_ represents oxygen vacancies).

Now we discuss the probable reason of band gap narrowing in TiO_2_ film with Fe doping, and widening in the case of Co doping after plasma treatment. As reported, the iron dopant acts as an acceptor impurity in TiO_2_ lattice^61^. Thus when the TiO_2_ film is doped with Fe, the acceptor levels of Fe along with oxygen vacancies are created in the band gap of TiO_2_^62^. In our case, as discussed above Ti^3+^ is also formed which creates energy level in the band gap, contributing to the reduction of band gap. Next, when this Fe doped TiO_2_ film was treated in air plasma, the Ti^3+^ levels and oxygen vacancies increases further with the treatment time, whereas no change in the dopant levels occurs as the dopant concentration was kept constant. The increase in Ti^3+^ levels and oxygen vacancies would further reduce the band gap of Fe doped TiO_2_ film. In case of Co doping, there is a formation of Co acceptor levels along with Ti^3+^ and oxygen vacancies levels in the band gap which reduces the band gap of Co doped TiO_2_ film. But when the film was treated with plasma we observed continuous widening in the band gap with treatment time. The observed increase in the band gap can be explained by Burstein-Moss effect. The probable reason for Burstein-Moss shift in this case is that with the treatment time the Ti^3+^ levels and oxygen vacancies increases more as compared to Fe doped case. By plasma treatment for 60 seconds the Ti^3+^ increases by 20%, oxygen vacancies increases by 15% in case of Fe doped TiO_2_, whereas Co doped TiO_2_ Ti^3+^ increases 30%, oxygen vacancies increases 25%. These created levels donate more electrons and thus shift the Fermi level to the conduction band, which increases the band gap of Co doped TiO_2_ film. The exact reason for this divergent behavior is unclear as of now but the most appropriate reason seems to us is, the on-site coulomb interaction/repulsion that are occurring only in case of Co doped TiO_2_ films^63^. When Co^2+^ ion substitutes Ti^4+^ ions, the imbalance positive charge inside the lattice is compensated by the formation of oxygen vacancies located near Co ion. The formation of oxygen vacancies is equivalent to the addition of two electrons per Co ion^64^,^65^. The oxygen vacancies produced in case of Co doped TiO_2_ thin films are higher as compared to Fe doped TiO_2_ films as observed by XPS. Suppose both Fe and Co doped films increase by same values of Ti^3+^ levels and oxygen vacancies, but due to Columbian interactions, which are only in case of Co doped TiO_2_^64^,^65^, the optical transition results in the blue shift of the absorption spectra. The proposed mechanism for both the Fe and Co doped TiO_2_ is illustrated in Fig. 10.

## Conclusion

Treatment by air plasma leads to significant change in the optical properties of TiO_2_ thin films. Unlike other treatment methods, in this approach the transparency of TiO_2_ thin film remains invariant. The charge separation centers i.e. oxygen vacancies and Ti^3+^ is created with the doping of metallic Fe and Co elements; however, they are significantly enhanced by the air plasma treatment. In Fe doped TiO_2_ thin film, the formation of oxygen vacancies and Ti^3+^ causes enhances absorbance and red shift due to the formation of energy levels in the band gap, whereas in Co doped TiO_2_ the Burstein-Moss shift is effective to make blue shift in the absorption spectra. Conclusively, we can say that the joint approaches i.e. low level/moderate doping and safe and low cost air plasma treatment resulted in enhanced optical properties of transparent TiO_2_ thin films, making them efficient candidate for transparent electrode applications.

## Experimental Methods

Thin films of TiO_2_, Fe doped TiO_2_ (Ti_0.95_Fe_0.05_O_2_) and Co doped TiO_2_ (Ti_0.95_Co_0.05_O_2_) were fabricated on glass substrate using dip-coating method. Titanium (IV) isopropoxide (TTIP, Ti[OCH (CH_3_)_2_]_4_, 97%, Aldrich) was used as precursor solution. First of all triethanolamine C_6_H_15_NO_3_, a stabilization agent was dissolved in C_2_H_5_OH, which resulted in a colorless solution. In this solution, the precursor solution Ti[OCH (CH_3_)_2_]_4_ was added dropwise to form a pale yellow solution with a continuous stirring. To avoid the precipitation of TiO_2_, C_2_H_5_OH and H_2_O was added in a ratio 9:1. Now during the sol gel synthesis the solutions of ferric nitrate (Fe (NO_3_)_3_.9H_2_O), and cobalt nitrate (Co (NO_3_)_2_.6H_2_O) were added separately as the dopant in TiO_2_. These solutions were stirred for two hours and allowed for ageing overnight. Then glass substrates cleaned with H_2_O, detergent, C_3_H_6_O and C_2_H_5_OH were coated with the aged solution. Coated films were dried and annealed at 400 °C to form transparent thin films. The fabricated films were treated in air plasma, generated in a vacuum coating unit (Hindhivac model: 12A4D), for varying treatment time; 0, 10, 30, and 60 seconds, respectively. The air plasma was generated at reduced pressure of 10^−3^ mbar in the vacuum chamber. During the treatment process the applied bias voltage was 30 volts with a power of 22.7 watt. After treating in plasma, the samples were analyzed for optical, structural, morphological and surface properties.

### Materials Characterization

The optical (absorbance, shift in absorption edge and band gap) properties of the films were studied by UV-Vis spectrophotometer (Perkin-Elmer Lambda 750). The band gap of Fe and Co doped thin films was calculated by using the absorbance spectra by plotting (*αhv*)^1/2^ against *hv*, where *hv* being incident photon energy. Surface morphology was studied using scanning electron microscopy (SEM), and elemental confirmation was done using energy dispersive X-ray (EDX). The structural analysis of the samples was done using X-ray diffractometer (XRD) (company name Rigaku, with Cu kα radiation, λ = 1.5406 Å), and to observe the effect of plasma treatment on surfaces states, X-ray photoelectron spectroscopy (XPS: VG Multilab 2000, Thermo electron corporation, UK) studies were performed.

## Additional Information

**How to cite this article**: Bharti, B. *et al*. Formation of oxygen vacancies and Ti^3+^ state in TiO_2_ thin film and enhanced optical properties by air plasma treatment. *Sci. Rep.* **6**, 32355; doi: 10.1038/srep32355 (2016).

## Supplementary Material

Supplementary Information

## Figures and Tables

**Table 1:**
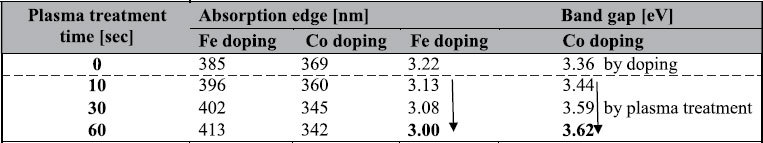
Variation in absorption edge and band gap of Fe and Co doped TiO_2_ thin films with plasma treatment time.
