# Supplementary material for: Formation of oxygen vacancies and Ti3+ state in TiO2 thin film and enhanced optical properties by air plasma treatment
Source: Sci Rep. 2016 Aug 30;6:32355. doi: 10.1038/srep32355 (PMC5004114; doi:10.1038/srep32355)
Supplement: Supplementary Information [file srep32355-s1.pdf]

Supplementary Information

Formation of oxygen vacancies and Ti^{3+} state in TiO_2 thin film and enhanced optical properties by air plasma treatment

Bandna Bharti[a], Santosh Kumar[b], Heung-No Lee[c] and Rajesh Kumar[a*]

[a]Jaypee University of Information Technology, Waknaghat, Solan-173234, H.P., India

[b]School of Materials Science and Engineering, Gwangju Institute of Science and Technology (GIST), 123 Cheomdangwagi-ro Buk-gu, Gwangju, 61005, South Korea

[c] Gwangju Institute of Science and Technology (GIST), 123 Cheomdangwagi-ro Buk-gu, Gwangju, 61005, South Korea

[*]Corresponding author's email: rajesh.kumar@juit.ac.in

The following materials provide additional information on the influence of air plasma treatment on surface and optical properties of TiO_2, Fe and Co doped TiO_2 thin films, and amount of oxygen in all samples obtained by XPS characterization.

SEM images of TiO_2, Fe and Co doped TiO_2 thin film with and without plasma treatment

Figure S1 (a-f) shows SEM images of pure TiO_2, Fe and Co doped TiO_2 thin films for different plasma treatment time. These images show films to be course, dense and composed of small grains (20-40nm). Also, from these images we observe that on the films surface there is no significantly change after treating them in plasma.

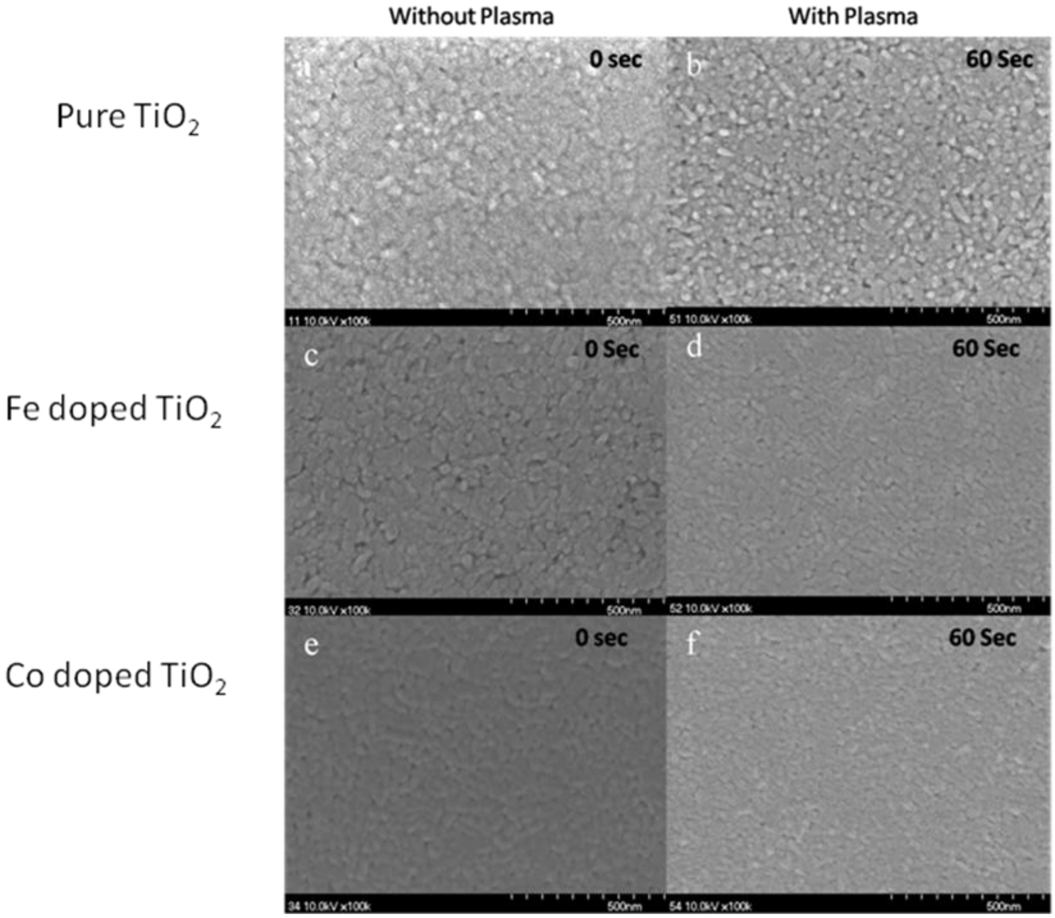

Figure S1: SEM images of TiO_2, Fe doped TiO_2 and Co doped TiO_2 thin films (a) pure TiO_2 film, (b) pure/treated TiO_2 film; plasma treatment time 60 seconds, (c) Fe doped/untreated TiO_2 film, (d) Fe doped/treated TiO_2 film; plasma treatment time 60 seconds (e) Co doped/untreated TiO_2 film, (f) Co doped/treated TiO_2 film; plasma treatment time 60 seconds.

X-ray diffractions of plasma treated TiO_2, Fe and Co doped TiO_2 thin films

XRD analysis was performed using X-ray diffractometer (XRD) (company name Rigaku, with Cu kα radiation, λ=1.5406 Å). Figure S2 represents XRD pattern of the films (pure TiO_2, Fe doped TiO_2, and Co doped TiO_2) which were treated for 30 seconds of treatment time. It reveals that the crystal structure of TiO_2 thin films remains unchanged after the different plasma treatment or the synthesis process.

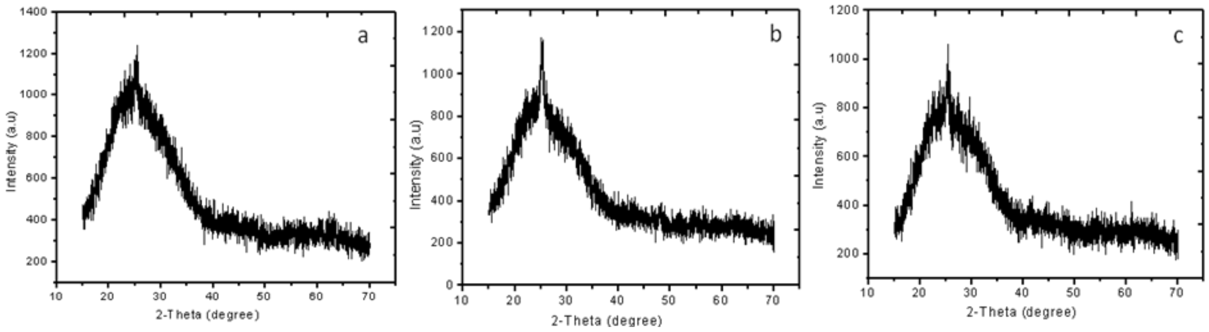

Figure S2: X-ray diffraction spectra of thin films for 30 seconds of plasma treatment (a) pure TiO_2, (b) Fe doped TiO_2, and (c) Co doped TiO_2 film.

Energy-dispersive X-ray analysis (EDX)

To detect the presence of doped elements Fe and Co, the films were investigated by EDX as shown in Figure S3. Figure S3(a) represents EDX spectrum of pure TiO_2 film. Here, the elements Ti and O are detected. Figure S3(b) is EDX spectrum of Fe doped TiO_2 film, in this spectrum element Fe with Ti and O is detected. Similarly, Figure S3(c) is EDX spectrum of Co doped TiO_2 film, containing Co element with Ti and O.

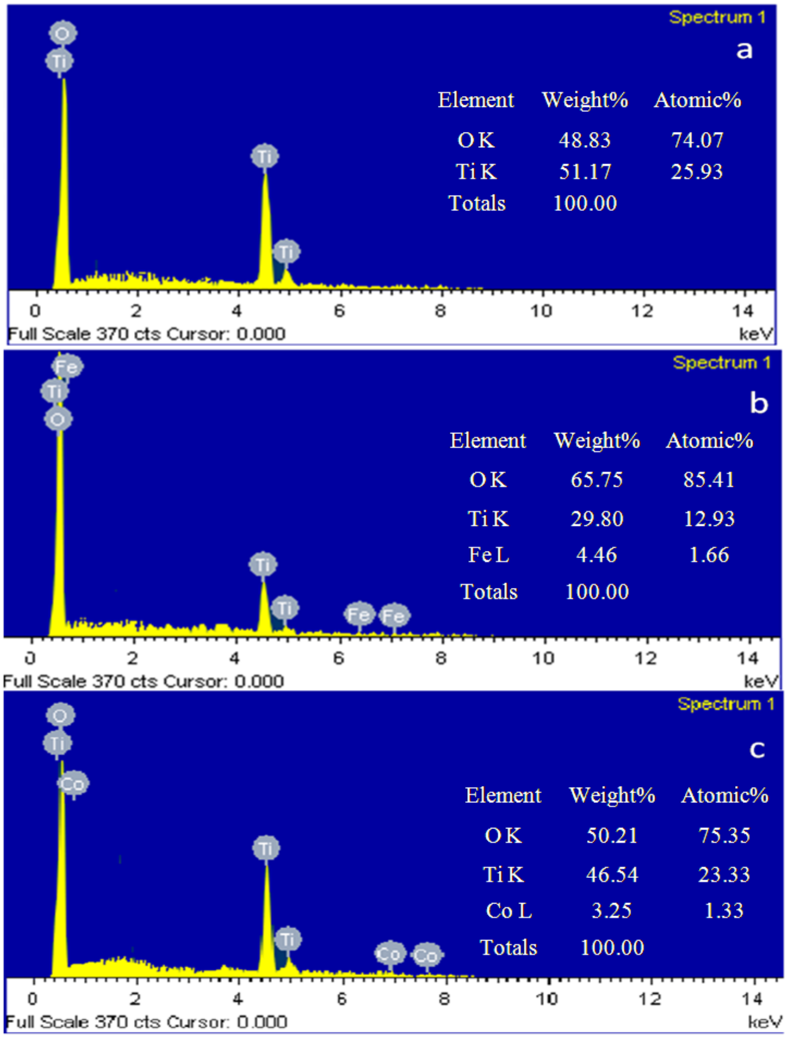

Figure S3: EDX spectrum of thin film of (a) pure TiO_2, (b) Fe doped TiO_2, and (c) Co doped TiO_2.

Determination of optical band gap:

The optical band gap of all the prepared TiO_2 films was calculated from the absorption spectrum using the Tauc relation:

$$(\alpha h\nu)^{1/2} = C(h\nu - E_g) \ldots\ldots.(1)$$

Where $h\nu$ is the incident photon energy, C is a constant and E_g represents optical band gap [1]. Absorption edge of the optical energy band gap of Fe and Co doped thin films was calculated using following formula:

$$E_{eV} = hc/\lambda \qquad (2)$$

Since the TiO_2 is an indirect band gap semiconductor; therefore, its band gap can be estimated from the plot of $(\alpha h\nu)^{1/2}$ versus $h\nu$ by extrapolating the straight portion of the graph to $h\nu$ axis as shown in Figure S4 and S5. The following figure represents absrobption spectra along with the Tauc plot of Fe doped TiO_2 films for increasing treatment time 0,10,30 and 60 seconds, reapectively. In these spectra the increasing absorbance can be seen with increasing treatment time.

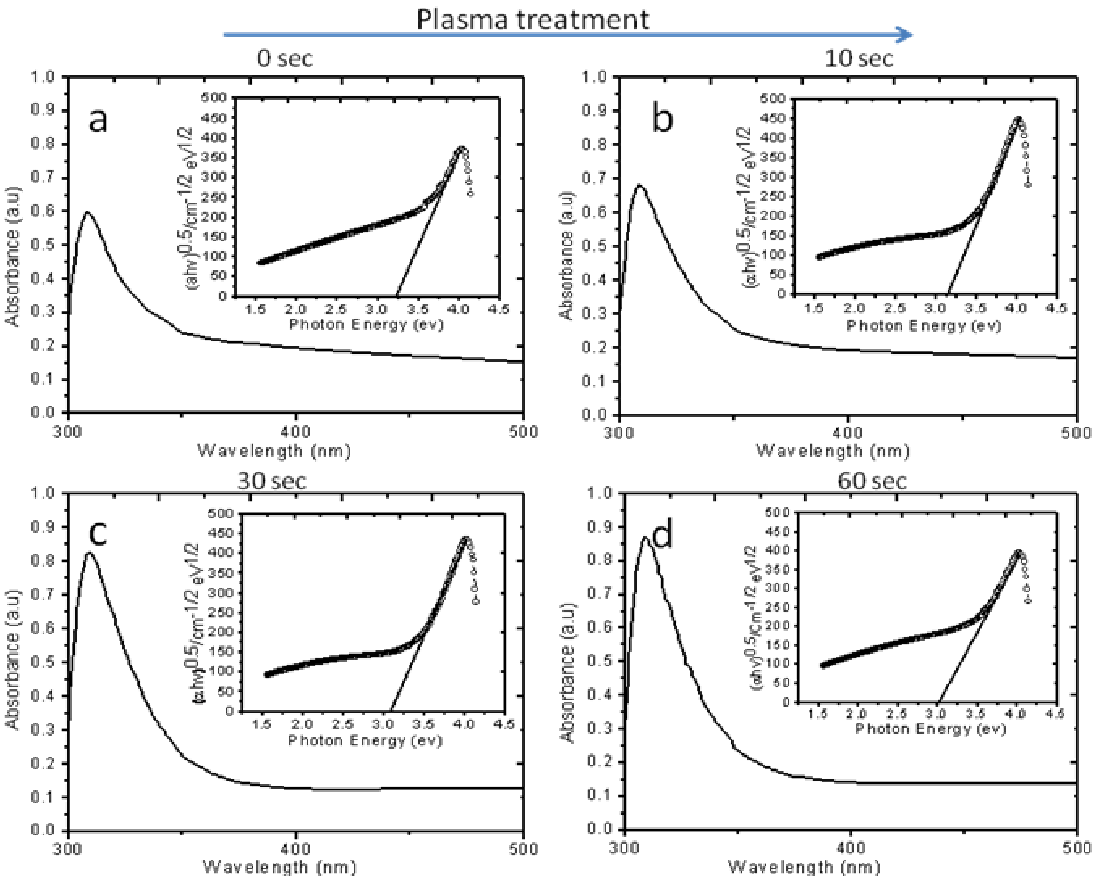

Figure S4. Optical absorption spectra and the inset shows $(\alpha h\nu)^{1/2}$ versus $h\nu$ plots for Fe doped TiO_2 thin film with and without air plasma treatment (a) 0 seconds, (b) 10 seconds, (c) 30 seconds, and (d) 60 sec.

The followig figure represents increasing band gap of Co doped TiO_2 film with increasing treatment time 0, 10,30 and 60, respectively.

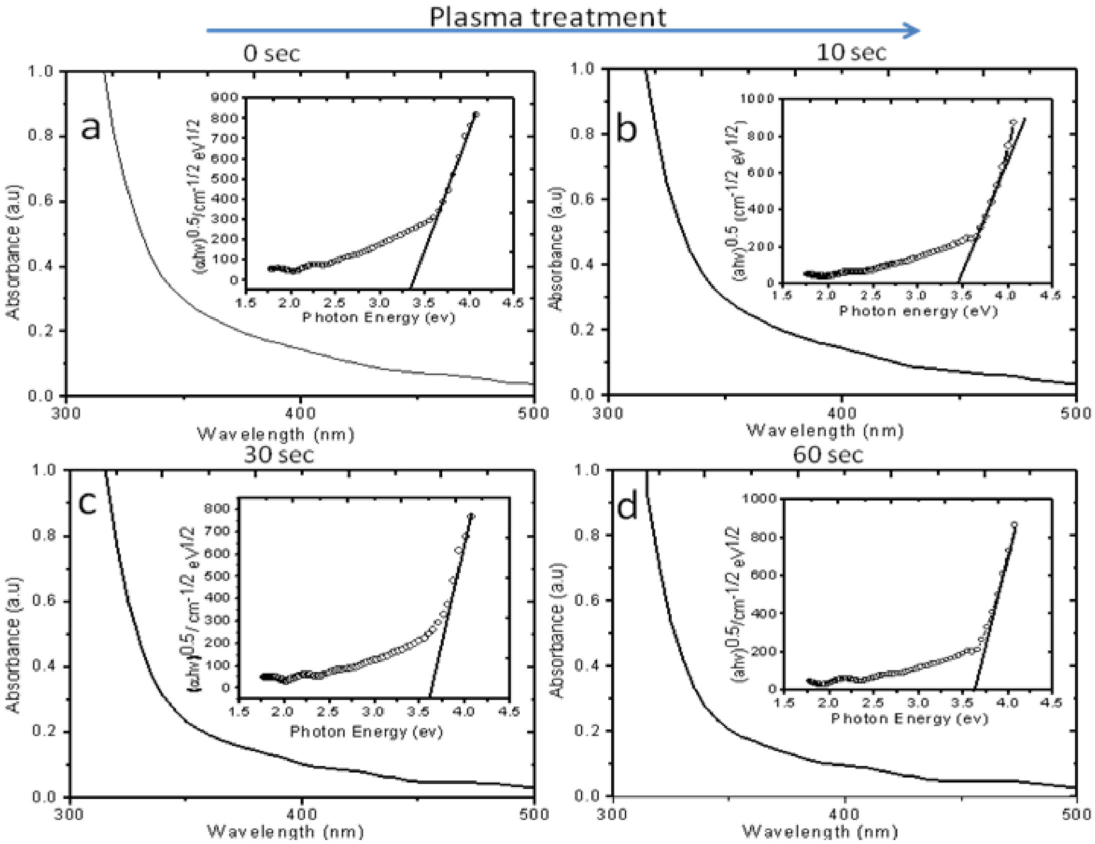

Figure S5. Optical absorption spectra and the inset shows$(\alpha h\nu)^{1/2}$ versus $h\nu$ plots for Co doped TiO_2 thin film with and without air plasma treatment (a) 0 seconds, (b) 10 seconds, (c) 30 seconds, and (d) 60 seconds.

X-ray photoelectron spectroscopy analysis (XPS)

XPS Analysis was performed using VG Multilab 2000, Thermo electron corporation, UK. The obtained XPS spectra deconvoluted with Gaussian shape. In Figure S6, we report the Ti2p peaks

for TiO_2, Fe doped TiO_2 and Co doped TiO_2 thin films. The XPS clearly reveals that there is a significant change in the Ti^{4+} state after the doping of Fe and Co and plasma treatment. There is also a change in the lattice oxygen and non lattice oxygen after plasma tretament.

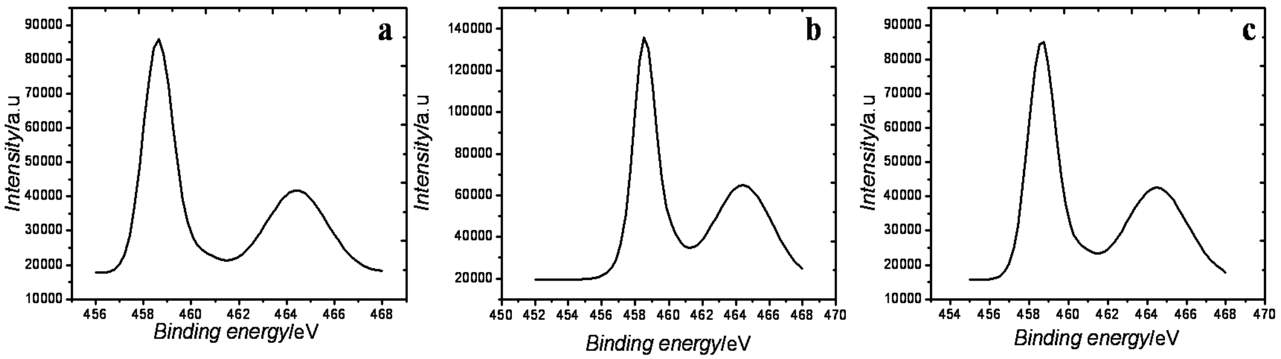

Figure S6: High-resolution XPS spectra of thin films Ti2p (a) TiO_2, (b) Fe doped TiO_2 (c) Co doped TiO_2.

Table S1. XPS data of different chemical states of O, Ti, Fe and Co elements on the surface of untreated TiO_2, Fe doped/treated $TiO_2$and Co doped/treated TiO_2 where O_L represents lattice oxygen, O_S represents sub oxide and O_V non lattice oxygen/ oxygen vacancies.

Sample	Plasma Treatment time (sec)	Ti^{4+}(eV)		Ti^{3+}(eV)		O(eV)			Total Percentage of Oxygen Vacancies
		Ti2p3/2	Ti2p1/2	Ti2p3/2	Ti2p1/2	O_L	O_S	O_V	
TiO_2	0	458.6	464.4	--	460.4	529.9	530.3	531.3	35
$Ti_{0.95}Fe_{0.05}O_2$	0	458.4	464.3	--	459.0	529.8	--	531.9	53.9
	60	458.5	464.4	--	459.0	529.7	--	531.5	61.9
$Ti_{0.095}Co_{0.05}O_2$	0	458.6	464.4	460.4	457.9	530.1	--	531.1	43.4
	60	458.5	464.3	460.6	457.4	530.1	--	531.8	54.2

Table S1 shows the binding energy of Ti2p and O1s for all the prepared samples with and

without plasma treatment. It is clear from the Table S1 that with the increase in the plasma treatment time Ti^{3+} state and the oxygen vacancies incrases. O_L represents lattice oxygen, O_S represents sub oxide and O_V non lettice oxygen/ oxygen vacancies. Table S2 shows peak position in the deconvulated spectra corresponding to Fe2p and Co2p

Table S2: XPS peak position of transition metal ions

Sample (Chemical Formula)	Plasma treatment time (seconds)	Binding energy of metal ions(eV)	
		2p3/2	2p1/2
$Ti_{0.95}Fe_{0.05}O_2$	0	710.1	724.6
	60	711.3	724.8
$Ti_{0.095}Co_{0.05}O_2$	0	781.0	796.9
	60	781.2	796.6

Supporting References:

[1] Yang, J. Y. *et al*. Grain size dependence of electrical and optical properties in Nb-doped anatase TiO_2. *Appl. Phys. Lett.* **95,** 0–3 (2009).
